# Genistein Attenuates Nonalcoholic Steatohepatitis and Increases Hepatic PPAR*γ* in a Rat Model

**DOI:** 10.1155/2015/509057

**Published:** 2015-07-13

**Authors:** Warinda Susutlertpanya, Duangporn Werawatganon, Prasong Siriviriyakul, Naruemon Klaikeaw

**Affiliations:** ^1^Department of Physiology, Faculty of Medicine, Chulalongkorn University, Bangkok 10330, Thailand; ^2^Department of Pathology, Faculty of Medicine, Chulalongkorn University, Bangkok 10330, Thailand

## Abstract

Nonalcoholic steatohepatitis (NASH) has become a global chronic liver disease, but no effective medicine has been proven to cure it. This study investigated the protective effects of genistein, a phytoestrogen, on NASH and examined whether it has any effect on hepatic PPAR*γ*. Male Sprague-Dawley rats were divided into four groups: control group fed ad libitum with standard rat diet, NASH group fed ad libitum with high-fat diet to induce NASH and NASH + Gen8 group and NASH + Gen16 group fed with high-fat diet plus intragastric administration of 8 or 16 mg/kg genistein once daily. After 6 weeks, liver samples were collected to determine MDA, TNF-*α*, PPAR*γ*, and histopathology. The findings were that levels of hepatic MDA and TNF-*α* increased in NASH group, but 16 mg/kg genistein reduced these levels significantly. Downregulation of hepatic PPAR*γ* was observed in NASH group, but genistein significantly upregulated the expression of PPAR*γ* in both NASH + Gen groups. The histological appearance of liver in NASH group presented pathological features of steatohepatitis which were diminished in both NASH + Gen groups. The results suggest that genistein attenuates the liver histopathology of NASH with upregulation of hepatic PPAR*γ*, reduction of oxidative stress, and inhibition of inflammatory cytokine.

## 1. Introduction

Nonalcoholic steatohepatitis (NASH) is a chronic liver inflammation caused by fat accumulation in hepatocyte. It is a subset of nonalcoholic fatty liver disease (NAFLD) that covers a spectrum ranging from benign hepatic steatosis to aggressive NASH which can progress to cirrhosis and hepatocellular carcinoma and eventually liver-related death [1]. NASH is usually present in the majority of patients with central obesity and diabetes mellitus that share insulin resistance as a common feature; therefore, it is also considered as a liver manifestation of metabolic syndrome [2]. With the rising prevalence of obesity in the developed world, NASH is becoming an increasingly important global problem. Unfortunately, no effective therapy has been proven to be against NASH [3]. Even though weight loss and lifestyle changes are the standard recommendations in overweight patients, it often fails and is not able to prevent NASH development [4].

However, nowadays many drugs have been tried in the treatment of NASH and one of the most frequently used therapeutic drugs is insulin-sensitizing drug, most of which are acting on peroxisome proliferators-activated receptor gamma (PPAR*γ*). PPAR*γ* is a ligand-dependent transcription factor that regulates fat metabolism, inflammation, cell differentiation, and apoptosis [5]. Increased expression of PPAR*γ* was observed in mice with hepatic steatosis induced by high-fat diet (HFD) [6]. On the other hand, its expression was decreased in HFD-induced NASH rats and correlated negatively with the severity of liver damage [7]. Accordingly, although the results of many studies are still controversial, PPAR*γ* may play an important role in the progression of NASH.

Genistein (4′,5,7-trihydroxyisoflavone), a phytoestrogen, is a main isoflavone found in soy. It is known as a tyrosine kinase inhibitor [8] and also has many health beneficial effects, for example, antioxidant, anti-inflammatory, and antifibrotic effects [9–11]. Furthermore, preventive effects of genistein on NASH have been shown in some studies [12, 13]. Nevertheless, it is not clear that genistein has any effect on hepatic PPAR*γ* expression in NASH model. Consequently, this study determined whether genistein could diminish pathological features of NASH induced by HFD and also affect PPAR*γ* expression in the liver.

## 2. Materials and Methods

### 2.1. Animal Preparation

Male Sprague-Dawley rats weighing 180–220 grams were purchased from the National Laboratory Animal Center, Mahidol University, Nakhon Pathom, Thailand. The animals were housed in a controlled temperature room at 25 ± 1°C with 12 h light/dark cycle and were given free access to food and water. The experimental procedure carried out on the animals was approved by the Ethics Committee of the Faculty of Medicine, Chulalongkorn University, Bangkok, Thailand (IRB approval number: 15/55).

### 2.2. Experimental Protocol

All rats were randomly divided into four groups. Group 1 (control, *n* = 8): rats were fed ad libitum with standard rat chow diet (containing 7% fat and 26% protein) purchased from Perfect Companion Group Co., Ltd. (Bangkok, Thailand) and they were given 0.1% dimethyl sulfoxide (DMSO) once daily by gavage tube for 6 weeks. Group 2 (NASH, *n* = 8): rats were fed ad libitum with lard-based HFD containing 81% fat and 4% protein for 6 weeks to induce NASH and also administered 0.1% DMSO orally as described in group 1. Group 3 (NASH + Gen8, *n* = 8): rats received ad libitum HFD plus intragastric administration of 8 mg/kg genistein (Cayman Chemical Company, MI, USA) dissolved in DMSO once daily for 6 weeks. Group 4 (NASH + Gen16, *n* = 7): rats were given ad libitum HFD and 16 mg/kg of genistein in 0.1% DMSO once daily by intragastric tube for 6 weeks. The amounts of genistein administered were based on the safe doses ranging from 1 to 16 mg/kg body weight according to the pharmacokinetic study of isoflavones by Busby and colleagues [14].

At the end of the experimental period, all rats were anesthetized with thiopental (50 mg/kg, intraperitoneal). The abdominal wall was opened, and the whole liver was rapidly removed and subsequently washed with cold normal saline. Two small pieces of the liver were frozen in liquid nitrogen and then stored at −80°C for measurements of malondialdehyde (MDA) and tumor necrosis factor-alpha (TNF-*α*). The remainder of liver was fixed in 40 g/L formaldehyde to determine histopathology and PPAR*γ* protein expression.

### 2.3. Hepatic Malondialdehyde (MDA) Determination

MDA level was quantified by using a commercial assay kit (Cayman Chemical, MI, USA) to examine thiobarbituric acid-reactive substances (TBARS). Briefly, frozen liver tissues were homogenized in radioimmunoprecipitation assay (RIPA) buffer with protease inhibitor on ice. Then the homogenates were centrifuged at 1,600 ×g for 10 minutes at 4°C and the supernatants were collected. After performing in accordance with the manufacturer's protocol, the absorbent of supernatants were measured at 540 nm and MDA concentrations were calculated from a standard curve. Moreover, the total protein contents were also measured to correct the MDA levels which were expressed in nmol/mg protein.

### 2.4. Assay of Liver Tumor Necrosis Factor-Alpha (TNF-*α*)

Portions of liver tissues were homogenized in RIPA buffer. Then liver homogenates were centrifuged at 12,000 ×g for 10 minutes at 4°C and the supernatants were collected. The amounts of TNF-*α* were measured by sandwich ELISA using a colorimetric commercial kit from R&D Systems (Minneapolis, MN, USA) according to the manufacturer's protocols. The total protein concentration of each sample was also required to correct the TNF-*α* level which was represented in terms of pg/mg protein.

### 2.5. Immunohistochemistry for Expression of Hepatic PPAR*γ*

After the liver samples were fixed in formaldehyde, they were embedded in paraffin and cut at 3 *μ*m. Next, tissue sections were deparaffinized and then retrieved the antigen in microwave. Afterward, the slides were incubated with 3% hydrogen peroxide to block endogenous peroxidase activity and then washed with phosphate buffer saline (PBS). Subsequently, the sections were incubated with mouse monoclonal antibody against PPAR*γ* (Santa Cruz Biotechnology, Santa Cruz, CA) at 1 : 50 dilution at 4°C overnight and washed again with PBS. After the tissues were incubated with the secondary antibody for 30 minutes at room temperature, the immunoreactivities were visualized by incubating the slides with diaminobenzidine (DAB) for 5 minutes. Finally, the sections were counterstained with hematoxylin. Under light microscopy, PPAR*γ* immunoreactive cells were identified as those with dark brown in their nuclei and digital images were taken in high magnification field from each sample. A thousand cells were counted manually for each rat and the numbers of positive stained cells were calculated and expressed as the percentage of immunoreactive cells. Quantification of immunostaining intensity was performed by measuring densitometry using ImageJ program (US National Institutes of Health, Bethesda, MD, USA).

### 2.6. Histopathological Examination

Paraffin-embedded liver tissues were sectioned and stained with hematoxylin and eosin. An experienced pathologist who was blinded to the experiment evaluated all samples. All histopathological changes were observed under light microscope. All fields in each section were examined for grading of steatosis, inflammation, and ballooning degeneration according to the criteria described by Brunt et al. [15].

The severity of steatosis was graded as the percentage of parenchymal cells containing fat as follows: 0 = less than 5% of hepatocytes containing fat, 1 = less than 33% of hepatocytes containing fat, 2 = 33–66% of hepatocytes containing fat, and 3 = more than 66% of hepatocytes containing fat.

Hepatic inflammation was scored from 0 to 3: 0 = no inflammation, 1 = mild focal zone 3 hepatocyte inflammation, 2 = moderate zone 3 hepatocyte inflammation, and 3 = severe zone 3 hepatocyte inflammation.

The presence of ballooning degeneration which is the key character used to distinguish the developed NASH from the less progressive forms of NAFLD was graded from 0 to 2: 0 = no ballooning cell, 1 = few balloon hepatocytes, and 2 = many balloon hepatocytes.

### 2.7. Statistical Analysis

Statistical analysis was performed by the Statistics Package for the Social Sciences (SPSS) software version 18.0 for Windows. Most data were presented as mean ± SD except for histopathological scores which were presented as frequency. Mean comparison among groups of animals was carried out with one-way analysis of variance (one-way ANOVA) followed by LSD post hoc test. Differences were considered statistically significant at *p* < 0.05.

## 3. Results

### 3.1. Effect of Genistein on Oxidative Stress Marker

**Figure 1:**
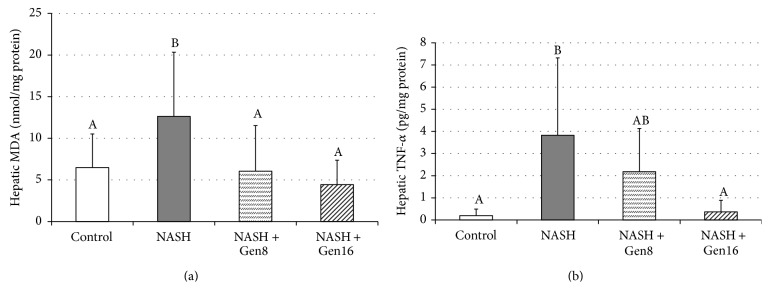
Effects of genistein on MDA (a) and TNF-*α* (b) in the liver of rats. Different superscript letters indicate statistically significant differences among groups (*p* < 0.05). Data are mean ± SD. Control (*n* = 8): rats fed with normal diet plus vehicle; NASH (*n* = 8): rats fed with high-fat diet (HFD) plus vehicle; NASH + Gen8 (*n* = 8): rats fed with HFD plus 8 mg/kg genistein; NASH + Gen16 (*n* = 7): rats fed with HFD plus 16 mg/kg genistein.

As shown in Figure 1(a), a significant increase of hepatic MDA level was observed in NASH group when compared with control group (12.63 ± 7.71 nmol/mg protein versus 6.48 ± 4.03 nmol/mg protein, *p* = 0.031). Conversely, genistein reduced the levels of MDA in NASH + Gen8 and NASH + Gen16 groups significantly (6.05 ± 5.48 nmol/mg protein in NASH + Gen8 group, *p* = 0.022, and 4.43 ± 2.94 nmol/mg protein in NASH + Gen16 group, *p* = 0.007).

### 3.2. Effect of Genistein on Inflammatory Cytokine

As shown in Figure 1(b), hepatic TNF-*α* level was elevated significantly in NASH group compared with the control (3.83 ± 3.50 pg/mg protein versus 0.19 ± 0.30 pg/mg protein, *p* = 0.002). On the other hand, the level of TNF-*α* in liver was significantly lower in NASH + Gen16 group than those in NASH group (0.36 ± 0.53 pg/mg protein versus 3.83 ± 3.50 pg/mg protein, *p* = 0.003). Although hepatic TNF-*α* level in NASH + Gen8 group also tended to be lower than those in NASH group, the difference was not statistically significant.

### 3.3. Effect of Genistein on PPAR*γ* Expression

**Figure 2:**
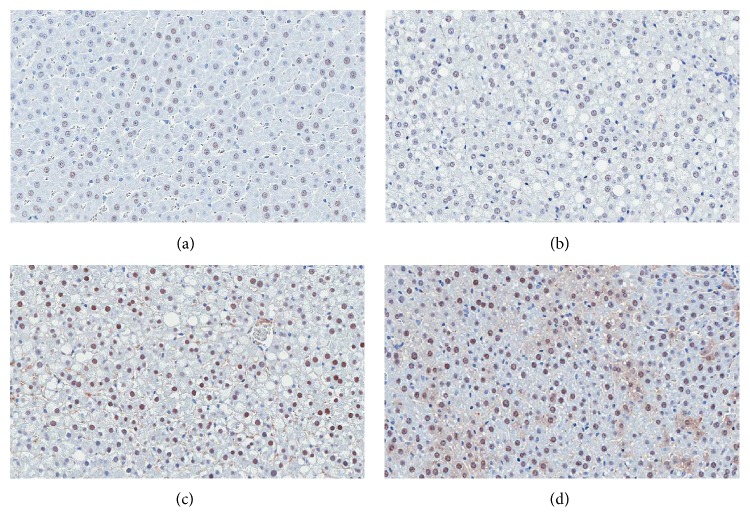
Effect of genistein on immunohistochemical staining of PPAR*γ* in the liver of rats. (a) Control group; (b) NASH group; (c) NASH + Gen8 group; (d) NASH + Gen16 group. Nuclear counterstaining was performed with hematoxylin. Positive stained cells contain dark brown nuclei. Images were obtained at ×200 magnification.

**Figure 3:**
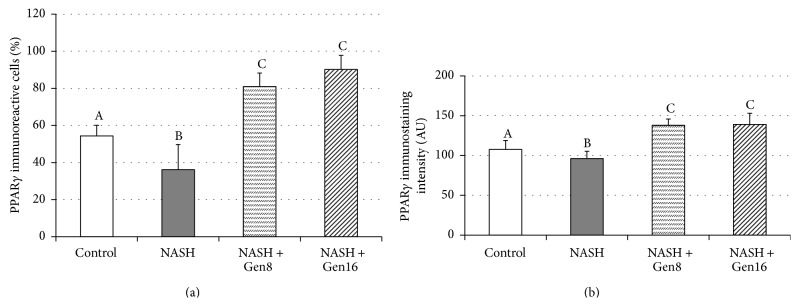
Effects of genistein on PPAR*γ* expression in the liver of rats. (a) The percentage of PPAR*γ* immunoreactive cells; (b) the immunostaining intensity of PPAR*γ*. Different superscript letters indicate statistically significant differences among groups (*p* < 0.05). Data are mean ± SD. Control (*n* = 8): rats fed with normal diet plus vehicle; NASH (*n* = 8): rats fed with high-fat diet (HFD) plus vehicle; NASH + Gen 8 (*n* = 8): rats fed with HFD plus 8 mg/kg genistein; NASH + Gen 16 (*n* = 7): rats fed with HFD plus 16 mg/kg genistein.

PPAR*γ* protein expression in the liver was studied by using immunohistochemical technique and PPAR*γ* positive stained cell was identified by dark brown nuclei (Figure 2). As shown in Figures 3(a) and 3(b), high-fat diet feeding significantly reduced the expression of hepatic PPAR*γ* in NASH group with the significant decreases in the percentage of PPAR*γ* immunoreactive cells and the immunostaining intensity when compared with the control group (36.20 ± 13.51% versus 54.34 ± 5.78%, *p* = 0.000, and 96.17 ± 9.30 versus 107.62 ± 11.30, *p* = 0.042, resp.). Nevertheless, PPAR*γ* expressions in the liver were enhanced by genistein in both NASH + Gen8 and NASH + Gen16 groups with the significant increases of PPAR*γ* immunoreactive cells (80.93 ± 7.36% in NASH + Gen8 group, *p* = 0.000, and 90.21 ± 7.57% in NASH + Gen16 group, *p* = 0.000). Moreover, densitometry analysis also showed the results similar to those of the percentages of PPAR*γ* positive stained cells (137.93 ± 7.83 in NASH + Gen8 group, *p* = 0.000, and 139.11 ± 14.04 in NASH + Gen16 group, *p* = 0.000).

### 3.4. Effect of Genistein on Liver Histopathology

**Figure 4:**
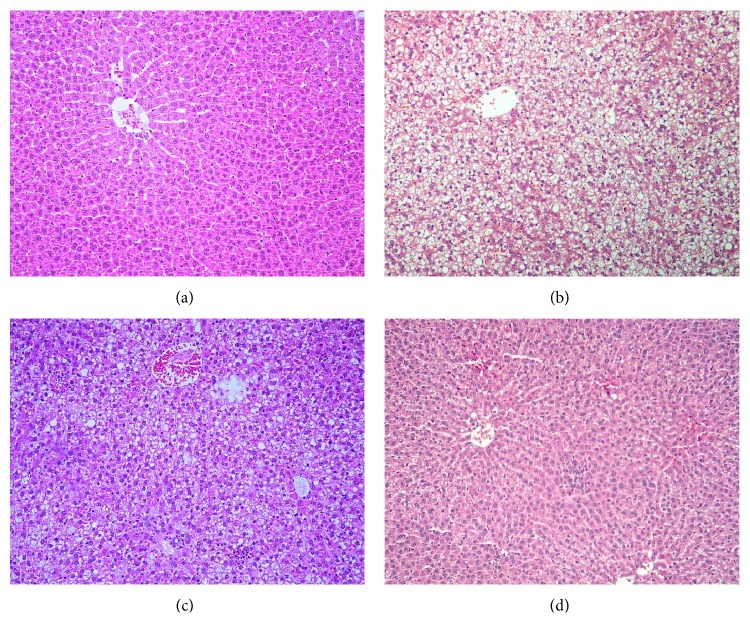
Effect of genistein on liver histopathology of NASH in rats. (a) Control group showed a normal structure of the liver; (b) NASH group presented steatohepatitis consisting of numerous fat vacuoles, inflammatory cells, and balloon cells; (c) NASH + Gen8 group showed mild macrovesicular steatosis and mild focal zone 3 inflammation; (d) NASH + Gen16 group maintained the normal structure with only minor changes. Liver sections were stained with hematoxylin and eosin. Images were obtained at ×100 magnification.

In control group, the liver sections represented normal histology as shown in Figure 4(a), whereas NASH group developed steatohepatitis, including macrovesicular steatosis, inflammatory cells infiltration, and ballooning degeneration in the liver (Figure 4(b)). In contrast, the histological appearance of liver sections in both NASH + Gen groups exhibited the improvement of steatosis and ballooning degeneration (Figures 4(c) and 4(d)). Especially in NASH + Gen16 group, all rats in this group had no liver inflammation. The summary of liver histopathological scores in all groups is shown in Table 1.

## 4. Discussion

At the present, it is recognized that NASH is one of the most common chronic liver conditions in general population. Even though the pathogenesis of NASH is not yet fully understood, the two-hit hypothesis [16] is the most widely accepted theory that describes the progression of NASH. With regard to this hypothesis, the build-up of fat in liver is the first hit causing hepatic steatosis that increases the susceptibility of liver to a variety of second hits, consisting of inflammatory cytokines, mitochondrial dysfunction, and oxidative stress, which contributes to liver necroinflammation and eventually fibrosis [17].

Although there is no definitive treatment for NASH, the benefits of genistein, a soy-derived isoflavone with antioxidant and anti-inflammatory activities, in the prevention of NASH have been studied in some research. Nonetheless, the effect of its on hepatic PPAR*γ* in NASH model has not yet been revealed. Moreover, the role of PPAR*γ* in NASH has remained contradictory. Therefore, we demonstrated the preventive effects of genistein against NASH and its effect on hepatic PPAR*γ*.

One of the processes of second hits that activates the transition of simple steatosis to NASH is oxidative stress. MDA, a marker for oxidative stress, is derived from lipid peroxidation which frequently arises in response to oxidative damage [18]. In fact, NASH patients had higher MDA level than healthy people [19] in accordance with our result that showed a significant increase of MDA level in NASH group as compared with normal control. However, this increase was alleviated by genistein, the greatest antioxidant among isoflavones [20]. Genistein acts as an antioxidant directly or indirectly by scavenging free radicals [21] or activating antioxidants [22].

Oxidative stress and lipid peroxidation produce many reactive oxygen species which can trigger production of various inflammatory cytokines, including TNF-*α* [23]. There is evidence suggesting that TNF-*α* plays a role in the evolution of NASH because the expressions of TNF-*α* and its receptor increase in patients with NASH compared to patients without NASH [24]. Corresponding to this result, we found an increase of TNF-*α* level in NASH rats but administration of 16 mg/kg genistein can reduce this increase significantly. The anti-inflammatory effect of genistein on NASH may be involved in suppression of c-Jun N-terminal kinase (JNK) and nuclear factor- (NF-) *κ*B pathways that leads to the inhibition of TNF-*α* synthesis [13].

It is known that PPAR*γ* is important to the prevention of NASH, although its role in NASH progression has rarely been described and some results are still unclear and contrary [6, 7]. Our result showed that hepatic PPAR*γ* protein expression was significantly dropped in HFD-induced NASH rats when compared with control rats; this result corresponds to the previous studies [25, 26]. This decreased PPAR*γ* expression in the liver of NASH group may be owing to an augmentation of TNF-*α* because TNF-*α* can inhibit PPAR*γ* activity at both pre- and posttranslational levels [27].

Interestingly, genistein significantly increased hepatic PPAR*γ* expressions in both NASH + Gen groups in this study. Likewise, another study also found an upregulation of PPAR*γ* in the liver of metabolic syndrome rats given genistein aglycone [28]. This may be associated with PPAR*γ* ligand property of genistein which can activate PPAR*γ* in a ligand-dependent manner [29, 30]. Additionally, it has been proved that overexpression of PPAR*γ* can attenuate NASH in mice by alleviation of biochemical and histopathological abnormalities. This PPAR*γ* effect could be due to downregulation of proinflammatory cytokines and upregulation of antioxidant as well as redistribution of fatty acid from liver to adipose tissue resulting in the reduction of hepatic steatosis [31, 32]. Therefore, it is possible that upregulation of PPAR*γ* by genistein in this study may contribute to the improvement of steatosis and necroinflammation in the liver sections of NASH + Gen rats.

Overall, the study revealed that feeding HFD for 6 weeks can cause NASH in Sprague-Dawley rats with the same histopathology of NASH as in human in the form of liver steatosis and necroinflammation. Meanwhile, we investigated the protective effects of genistein, which can activate PPAR*γ*, on NASH induced by high-fat diet and found that hepatic PPAR*γ* expression was decreased in NASH rats but genistein upregulated its expression as well as diminished liver oxidative stress and inflammation. These appeared to have contributed to the improvement of liver histopathology of NASH. Further research is required to clarify the molecular mechanism behind the effect of genistein on hepatic PPAR*γ* in NASH model, such as the activation of JNK and NF-*κ*B signalling pathways, and the expression of PPAR*γ* target genes in the liver.

## 5. Conclusions

In summary, our results show an anti-NASH effect of genistein which is probably related to the increase of PPAR*γ* expression in the liver. From the findings, it is suggested that genistein effectively attenuates the emergence of NASH, and this may be useful for further studies and applications for NASH protection.

## Figures and Tables

**Table 1:** Summary of histopathological scores in all groups.

Group	*n*	Steatosis				Inflammation				Ballooning		
		0	1	2	3	0	1	2	3	0	1	2
Control	8	8	—	—	—	8	—	—	—	8	—	—
NASH	8	—	7	—	1	4	2	2	—	—	2	6
NASH + Gen8	8	2	4	1	1	3	3	2	—	2	1	5
NASH + Gen16	7	4	2	1	—	7	—	—	—	3	4	—

Data are expressed as the number of rats presenting each score of histopathology. Levels of steatosis: 0 = <5% of hepatocytes containing fat; 1 = <33% of hepatocytes containing fat; 2 = 33–66% of hepatocytes containing fat; 3 = >66% of hepatocytes containing fat. Levels of inflammation: 0 = normal; 1 = mild; 2 = moderate; 3 = severe. Levels of ballooning degeneration: 0 = no ballooning; 1 = few balloon cells; 2 = many balloon cells.

## Abbreviations

DAB: Diaminobenzidine
DMSO: Dimethyl sulfoxide
HFD: High-fat diet
JNK: c-Jun N-terminal kinases
MDA: Malondialdehyde
NAFLD: Nonalcoholic fatty liver disease
NASH: Nonalcoholic steatohepatitis
PBS: Phosphate buffer saline
PPAR*γ*: Peroxisome proliferators-activated receptor gamma
RIPA: Radioimmunoprecipitation assay
TBARS: Thiobarbituric acid-reactive substances
TNF-*α*: Tumor necrosis factor-alpha.
